# Development of a prototype antenna radiation pattern measurement system using Software-Defined Radio

**DOI:** 10.1016/j.ohx.2025.e00695

**Published:** 2025-09-06

**Authors:** Alex Kana-Chuctaya, Alexander Hilario-Tacuri

**Affiliations:** Universidad Nacional de San Agustín de Arequipa, Arequipa, Peru

**Keywords:** Antenna parameters, Graphical user interface, Low-cost, Radiation pattern, Raspberry Pi, Software Defined Radio

## Abstract

This work presents the design and implementation of an automated prototype system for measuring antenna radiation patterns, using Software Defined Radio (SDR), a stepper motor-driven rotational platform, and custom 3D printed components. The system is powered by a Raspberry Pi processing unit, equipped with a touchscreen interface for real-time control and data visualization. The prototype enables automated 360°sweeps in either the horizontal (azimuth) or vertical (elevation) plane, facilitating signal strength measurements across a broad sub-6 GHz frequency range (70 MHz – 5.9 GHz). The prototype was validated by measuring the radiation pattern of an ultra-wide band (700 to 6000 MHz) flexible antenna under far-field conditions and in non-anechoic environment, demonstrating its practical applicability with acceptable accuracy. Performance was evaluated by comparing the measured radiation patterns against the manufacturer’s reference data, yielding a root mean square error (RMSE) and a mean absolute error (MAE) below 0.172 (3.260 dB) and 0.139 (2.625 dB), respectively. These results indicate that the prototype offers a low-cost, reliable, modular, and adaptable solution for antenna characterization, suitable for both academic research and practical telecommunications applications. Furthermore, the hardware and software are open source, promoting ease of replication and enabling future enhancements.

## Graphical abstract

**Figure:**
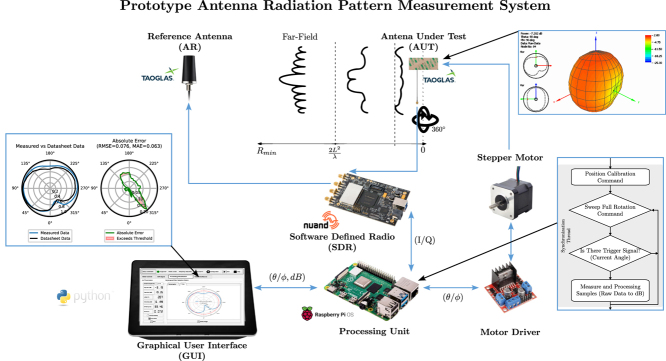

## Specifications table

**Table:** 

Hardware name	Prototype antenna radiation pattern measurement system
Subject area	• Educational tools and open source alternatives to existing infrastructure

Hardware type	• Measuring physical properties and in-lab sensors

Closest commercial analog	MegiQ RMS-0460 (370 MHz to 6 GHz Radiation Measurement System)

Open source license	GNU General Public License v3.0 (GPL-3.0), CERN Open Hardware License Version 2 - Permissive (CERN OHL-P v2) and Creative Commons Attribution 4.0 International (CC BY 4.0) licenses.

Cost of hardware	US $ 989

Source file repository	https://osf.io/kbm3f

## 1 Hardware in context

Antennas are fundamental components of telecommunications systems, enabling the transmission and reception of electromagnetic waves. Their performance directly impacts the efficiency and reliability of various telecommunications applications, including wireless communication networks, satellite systems, radars, and emerging 5G technologies. Among key performance metrics, the radiation pattern is particularly critical, as it defines the antenna’s directional characteristics and overall efficiency [1], [2]. Accurate measurement of the radiation pattern is essential to verify that antennas meet design specifications and operate effectively in their intended operational environments. Traditionally, characterizing antenna radiation patterns requires the use of vector network analyzers (VNAs) in controlled environments such as anechoic chambers. These systems are capable of measuring parameters such as gain, directivity, efficiency, and half-power beamwidth (HPBW) with high accuracy [3]. Despite their accuracy, conventional measurement setups present notable limitations: they are expensive, often inaccessible to smaller institutions, and involve complex operation. With the rapid deployment of 5G networks and the proliferation of Internet of Things (IoT) devices, there is an increasing demand for efficient, cost-effective solutions for antenna design and characterization. Although proprietary commercial systems, such as the MegiQ RMS-0460 radiation measurement [4], which supports measurements from 370 MHz to 6 GHz, offer robust functionality, they remain financially prohibitive for many, particularly in academic and small-scale research environments. For instance, the RMS-0460 is priced at approximately $18,560, making it inaccessible to a wide range of users seeking scalable and flexible testing solutions.

Software-defined radios (SDRs) have revolutionized the field of telecommunications by replacing traditional hardware-based functionalities with flexible, software-driven architectures. These devices are capable of both transmitting and receiving signals across wide frequency ranges, and their behavior is governed by programmable algorithms [5], [6]. The growing cost-effectiveness and accessibility of SDR platforms makes have made them an attractive alternative for antenna measurement applications, offering key advantages such as cost-effectiveness, reconfiguration, and portability. As a result, several SDR-based studies have explored the implementation of SDRs for antenna characterization (specifically through radiation pattern measurement), with the goal of replicating the performance of conventional systems. However, existing SDR-based approaches often exhibit notable limitations. Some focus primarily on near-field measurements, which are not directly applicable to far-field performance analysis [7], [8], [9]. Others, while open-source, lack comprehensive documentation and reproducibility [10]. Additionally, certain implementations rely on platforms such as GNU Radio Companion, which, despite their accessibility, impose constraints on system flexibility and real-time control [11], [12], [13].

SDR-based systems present a promising opportunity to democratize access to advanced measurement tools, thereby fostering innovation in antenna design for modern telecommunications applications. Despite their inherent advantages, SDRs still face significant challenges in achieving the measurement accuracy typically associated with VNAs. These challenges include noise and interference, signal processing complexity, and limitations in resolution and the supported frequency range [14]. Overcoming these challenges is essential for the development of a viable SDR-based solution for antenna measurement.

Taking into account the pros and cons of using SDRs and the importance of the antenna design process, this work proposes the design and implementation of a low-cost prototype system for measuring antenna radiation patterns using SDRs. The system leverages digital signal processing techniques to enhance measurement precision, with the objective of approaching the accuracy levels of traditional VNA-based methods. The prototype also incorporates a graphical user interface (GUI) that facilitates automated operation and visualization in real-time, further enhancing usability and accessibility.

## 2 Hardware description

### 2.1 Measurement methods

Antenna characterization is commonly performed using two established measurement techniques: near-field and far-field methods. The distance between the transmitting and receiving antennas is a crucial factor, as the radiated electromagnetic field distribution varies significantly between these regions. In a far-field measurement system, the antenna under test (AUT) is irradiated by a uniform plane wave, allowing direct evaluation of parameters such as gain, directivity, and radiation pattern. Conversely, near-field measurement systems, involve sampling the electromagnetic fields in close proximity to the antenna using a probe. The far-field characteristics are then derived computationally through near-field to far-field transformations [15], [16].

In this work, the antenna measurement approach is based on the far-field method. The far-field region is typically defined as beginning at a minimum distance $R_{min}$ (m) given by

(1) $$R_{min}=\frac{2L^{2}}{\lambda }$$

where L and λ are the antenna length (m) and the wavelength (m), respectively.

### 2.2 Prototype system design

**Fig. 1:**
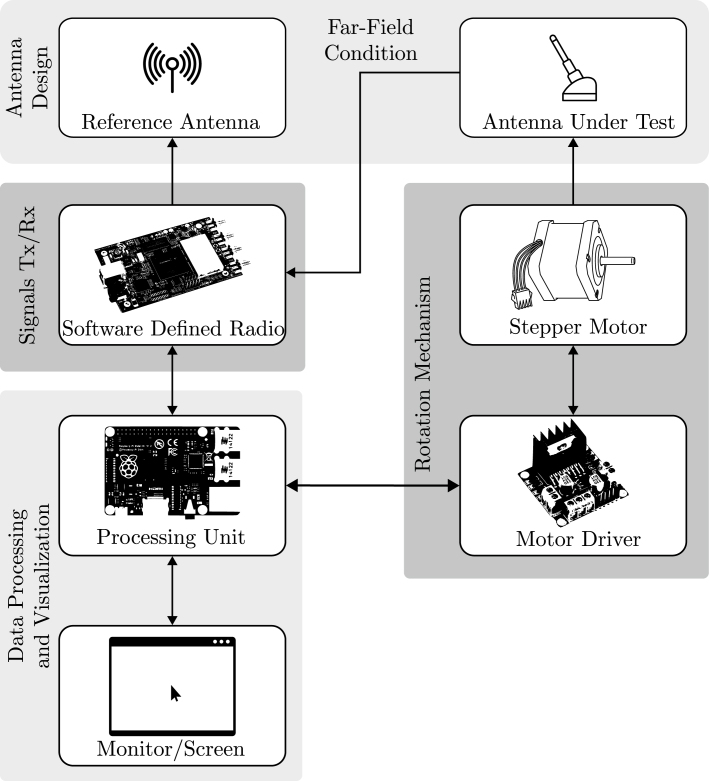
Block diagram of the proposed prototype.

The proposed block diagram for the development of the SDR-based antenna measurement prototype is presented in Fig. 1. The system is composed of several interconnected modules, beginning with user interaction through the data processing and visualization module. This central module manage both the control of the rotation mechanism and the transmission and reception of signals via the SDR. Signal data from the SDR is automatically transmitted and acquired by the antenna design module, where it is processed and visualized in real time. A detailed description of each module is provided below.

The data processing and visualization module is composed of two submodules: a processing unit and a display interface. These components were selected based on criteria such as versatility, broad community support, compatibility with other modules, and overall cost-effectiveness. This configuration enables the development of computationally efficient applications and GUIs using the Python programming language in conjunction with the PyQt5 library. In the proposed system, a Raspberry Pi 4B serves as the processing unit, while a 7-inch touchscreen display is employed for user interaction and real-time visualization. The rotation mechanism module comprises two submodules: the stepper motor and its corresponding drive. These components were selected for their ability to provide accurate positional feedback, enable precise angular control, and support the required current levels for stable operation. In this work, a NEMA 17 stepper motor with a step resolution of 0.9° is employed to facilitate controlled rotation, while an L298N motor driver is used as a controller of the stepper motor. The transmission and reception signal module consists mainly of an SDR, selected from a wide range of commercially available devices based on the specifications outlined in Table A.1. The selection criteria included key factors such as performance, flexibility, broad frequency coverage, high bandwidth, full-duplex capability, and overall cost-effectiveness. In this work, the bladeRF 2.0 micro xA4 SDR was selected due to its superior features and advantages compared to other available SDRs. Finally, the antenna design module comprises both the reference antenna (AR) and the antenna under test (AUT) submodules. The selection of these antennas is based on the wide frequency range (sub-6 GHz) supported by the selected SDR, the availability of manufacturer-provided data (such as radiation pattern and other key performance parameters), and their cost-effectiveness. In this work, the Shockwave 600–6000 MHz permanently mounted external antenna was selected as the AR, while the Maximus 700–6000 MHz ultra-wideband flexible antenna was employed as the AUT.

The integration of the selected components is designed to be straightforward and efficient. To facilitate this, 3D-printed parts were employed for the structural support of both the rotation mechanism and the antenna design module (AR and AUT). These parts were fabricated using polyethylene terephthalate glycol (PETG), a material chosen for its enhanced mechanical strength and durability. It is important to note that the prototype system is developed as an open-source system, allowing for future adaptation and improvement. Consequently, the implementation is not strictly limited to the specific components employed in this work. Based on the selected configuration, the total cost of the implementation is approximately $ 989, as detailed in Table 3. Despite this moderate cost, the system offers a cost-effective alternative to commercial solutions that rely on high-end, proprietary equipment, positioning the prototype as a viable low-cost alternative solution for antenna measurement applications.

### 2.3 Important aspects

The prototype proposed in this work:

• Serves as a validation tool for antenna design, specifically by measuring its radiation pattern within the range of 70 to 5999 MHz.
• It is low-cost, open source solution in comparison to conventional commercial proprietary systems that utilize high-end equipment such as VNAs. While some commercial antenna measurement systems can be built using RF signal generators and spectrum analyzers, resulting in lower costs, they still tend to be relatively expensive compared to the increasing demand for more affordable solutions within the sub-6 GHz frequency range.
• Facilitates the compensation of measurement errors through the application of signal processing and post-processing techniques, thereby eliminating the need for anechoic chambers.
• Feature a simple and automated operation facilitated by an intuitive GUI.

## 3 Design files summary

### 3.1 Hardware files

• Transmitting antenna support: This structure provides a fixed housing for the AR, enabling the transmission of a continuous wave (CW) signal. It offers sufficient space to accommodate the antenna’s SMA connector cable and allows for height adjustment based on the size of the selected antenna. The support is 3D printed, and the design files for printing are available in .stl format.
• Receiving antenna support: This structure accommodates the housing for both the stepper motor and its drive, as well as the rotation platform. The design of the rotating platform facilitates the orientation of the AUT both horizontally and vertically via an adjustment mechanism, allowing for manual adjustments based on the selected antenna. Additionally, this support is 3D printed, and the design files for printing are available in .stl format.
• Wiring diagram: This file provides a detailed representation of the electrical connections among the submodules that comprise the prototype system, presented in a modifiable wiring diagram format with a .fzz extension.

These hardware files have been developed in Solidworks 2021 software. They are documented and the .stl format files can be accessed in Table 1.

**Table 1:** List of hardware design files.

Design filename	File type	Open source license	Location of the file
Transmitting antenna support (part 1)	3D model (.stl)	CERN OHL	https://osf.io/ynr5k
Transmitting antenna support (part 2)	3D model (.stl)	CERN OHL	https://osf.io/atsu8
Receiver antenna support (part 1)	3D model (.stl)	CERN OHL	https://osf.io/kpmu6
Receiver antenna support (part 2)	3D model (.stl)	CERN OHL	https://osf.io/4jpuf
Receiver antenna support (part 3)	3D model (.stl)	CERN OHL	https://osf.io/8jtch
Receiver antenna support (part 4)	3D model (.stl)	CERN OHL	https://osf.io/c7v6p
Receiver antenna support (part 5)	3D model (.stl)	CERN OHL	https://osf.io/39kdn
Wiring diagram	Fritzing (.fzz)	CERN OHL	https://osf.io/y35nc

### 3.2 Software files

• Main code: This file imports all modules and process threads, creates instances, and configures parameters such as the GUI size and the utilization of scrollbars.
• Modules: This section includes the classes that comprise the GUI, encompassing the configuration of SDR connectivity; control of parameters for both the rotation mechanism and SDR signals (transmission and reception); as well as the processing, post-processing, visualization of the radiation pattern, and validation of the results.
• Threads: This section encompasses the threads designated for parallel processing, including SDR transmission and reception processing, rotation control, and the synchronization of angle and signal power data.
• Central storage: This section contains the initial configuration of the SDR and facilitates read and write access from any module.

These software files are documented with a .py extension and can be accessed in Table 2.

**Table 2:** List of software files.

Design filename	File type	Open source license	Location of the file
Main code	Python (.py)	GNU GPL	https://osf.io/8r45m
Module (connect configuration)	Python (.py)	GNU GPL	https://osf.io/qbc8k
Module (motor control)	Python (.py)	GNU GPL	https://osf.io/y7hjd
Module (sdr signal processing)	Python (.py)	GNU GPL	https://osf.io/m9hse
Module (radiation pattern)	Python (.py)	GNU GPL	https://osf.io/ae7jb
Module (validation results)	Python (.py)	GNU GPL	https://osf.io/y69zq
Thread (transmission)	Python (.py)	GNU GPL	https://osf.io/tayvh
Thread (receiving)	Python (.py)	GNU GPL	https://osf.io/yv8sd
Thread (rotation control)	Python (.py)	GNU GPL	https://osf.io/huan5
Thread (synchronization process)	Python (.py)	GNU GPL	https://osf.io/r5n8d
Central storage (sdr configuration)	Python (.py)	GNU GPL	https://osf.io/kepdj

## 4 Bill of materials summary

The list of components, along with their prices and reference sources is documented and can be found in Table 3.

**Table 3:** List of components with price and references.

Designator	Component	Number	Cost per unit - currency	Total cost - currency	Source of materials	Material type
Software Defined Radio	bladeRF 2.0 micro xA4	1	US $ 560	US $ 560	https://rb.gy/jkijfl	Other
Processing Unit	Kit Raspberry Pi 4B 4Gb	1	US $ 115	US $ 115	https://rb.gy/500gm5	Other
Screen	Screen $7^{\prime \prime }$ Raspberry Pi	1	US $ 85	US $ 85	https://rb.gy/d37dvt	Other
Case	Case (Pi 4B) for The $7^{\prime \prime }$ Touchscreen	1	US $ 30	US $ 30	https://rb.gy/skqpng	Other
Motor Driver	H-Bridge L298N	1	US $ 4	US $ 4	https://rb.gy/pbxmzi	Other
Stepper Motor	Bipolar 0.9 deg Nema 17	1	US $ 20	US $ 20	https://rb.gy/vbrxa9	Other
Mechanical Parts	3D Tx/Rx Antenna Support	1	US $ 90	US $ 90	–	Other
Reference Antenna (AR/Tx)	Taoglas TLS.01.305111	1	US $ 75	US $ 75	https://rb.gy/e7myp4	Other
Antenna Under Test (AUT/Rx)	Taoglas FXUB66.01.0150C	1	US $ 10	US $ 10	https://rb.gy/qzsjvp	Other

## 5 Build instructions

### 5.1 System architecture design

**Fig. 2:**
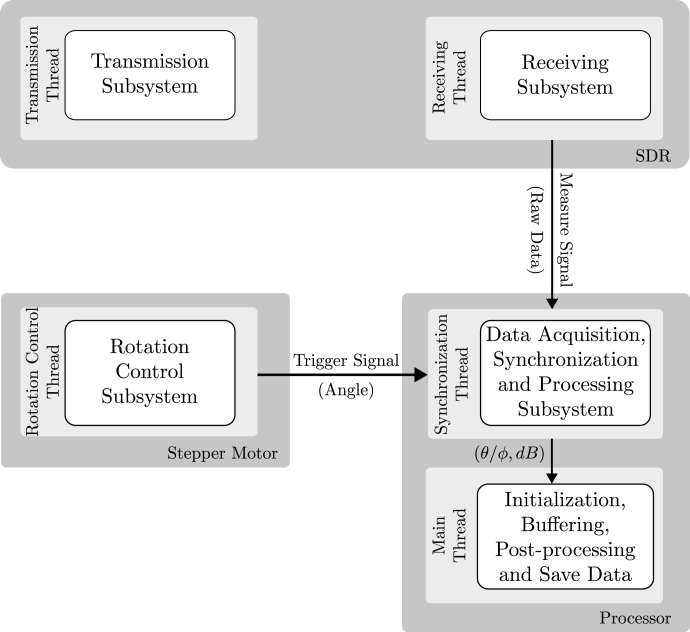
Architecture of the proposed prototype.

The architecture of the proposed prototype system is illustrated in Fig. 2, which depicts the processing threads of the subsystems operating in parallel, as well as the data flow for both angle and signal power. The threads associated with the SDR transmission and reception subsystems operate continuously. When an angle trigger signal is received from the rotation control subsystem thread, the synchronization and processing subsystem thread captures the corresponding signal measurement for that angle (θ/ϕ) and converts it into decibel (dB) power values. This pair of values (θ/ϕ,dB) is then plotted in both rectangular and polar forms in real time. Subsequently, this data undergoes post-processing and is stored within the main thread of the system.

### 5.2 Mechanical assembly

**Fig. 3:**
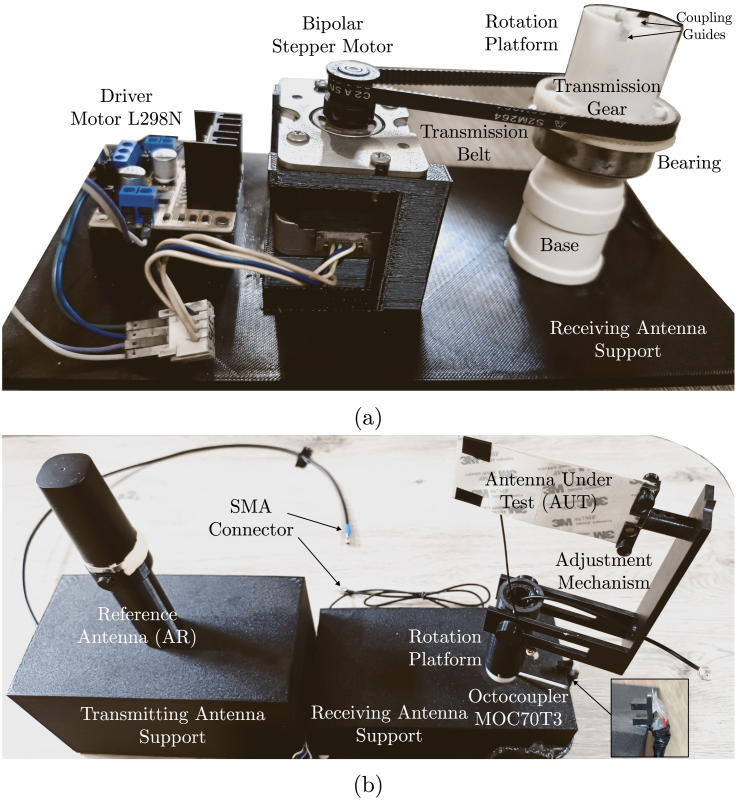
The mechanical assembly process takes place inside and outside the support structure. (a) Internal mechanical assembly and (b) external mechanical assembly.

The base structure comprises the supports for both the transmitting and receiving antennas, as detailed in Table 1. These supports are fabricated using PETG through 3D printing services. Subsequently, the NEMA 17 stepper motor, its L298N driver, and the rotation platform are attached to the receiving antenna support utilizing bolts, nuts, screws, and adhesive, respectively. The rotary platform is composed of a base, a bearing, a transmission gear and an adjustment mechanism, that are assembled with adhesive and coupling guides. The stepper motor is coupled to the rotating platform via a drive belt and the transmission gear, establishing a rotation ratio of 3.6:1 between the stepper motor and the rotating platform. A reference sensor, specifically the octocoupler MOC70T3, is installed to calibrate the 0° angle using silicone on the receiving antenna mount. Finally, the AR and AUT antennas are positioned at their designated locations on both the transmitting and receiving antenna supports. Fig. 3 illustrates the processes of internal and external mechanical assembly. The system is now prepared for electrical connections.

### 5.3 Electrical assembly

**Fig. 4:**
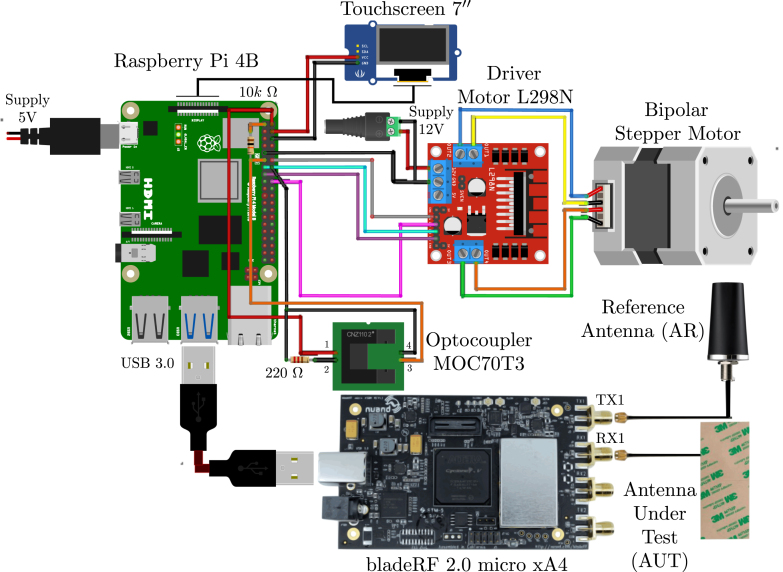
Wiring diagram of the proposed prototype.

Once the base structure is complete, the installation of the drive wiring for the stepper motor is initiated. Additionally, the driver, reference sensor, and 7-inch touchscreen are connected to the general-purpose input and output (GPIO) pins of the Raspberry Pi. The bladeRF device is subsequently connected to the Raspberry Pi using a USB 3.0 cable, and the necessary drivers and firmware are installed as described in the installation guide (https://osf.io/zh2es). Following this, the AUT is connected to the RX input of the SDR, while the TX output of the SDR is connected to the AR using SMA cables and connectors, ensuring compatibility with the corresponding channels for full-duplex operation. Finally, the 12 V power supply is connected to the motor controller, and the 5 V power supply is connected to the Raspberry Pi. This entire process is illustrated in Fig. 4, and the corresponding wiring diagram file, with a .fzz extension, can be accessed in Table 1. The system is now prepared for software assembly.

### 5.4 Software assembly

**Fig. 5:**
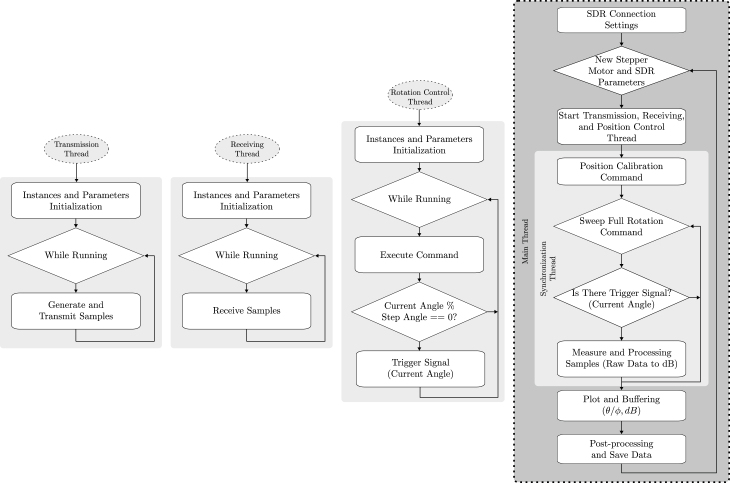
Flowchart illustrating the logic of the proposed prototype.

The flowchart illustrating the programming of the prototype system is presented in Fig. 5, and the files associated with these process threads are available in Table 2. This flowchart delineates the logical sequence of the main program and the parallel operation of the process threads. The sequence commences with the configuration of the SDR, during which the device’s serial number, operational mode, and the designated channel are established. The system subsequently accepts new values entered via the GUI for the parameters pertaining to both the SDR and the stepper motor. Following this, the additional threads are initiated and operate concurrently until the completion of the main program.

Once the transmission process, SDR reception, and platform rotation control threads are initiated, the synchronization thread will start, wherein reference position calibration and a 360° sweep are conducted. During the sweep, if a trigger signal is detected corresponding to the predetermined step angle, the current angle is recorded, and the measurement and processing of samples received from the reception threads are performed. This process involves measuring the raw signal multiple times at the current angle (θ/ϕ) and converting this measurements into power values (in dB), which are subsequently averaged. Finally, this pair of values (θ/ϕ,dB) is plotted, post-processed, and stored. A detailed description of each thread follows.

#### 5.4.1 Transmission thread

In this thread, the instances and parameters related to the transmission are initialized. This thread operates continuously, generating and transmitting the baseband samples. The generated baseband signal $s_{bb}\left(t\right)$ is a complex signal represented in the form of I/Q (in-phase and quadrature components) given by

(2) $$s_{bb}\left(t\right)=I\left(t\right)+jQ\left(t\right).$$

For a constant wave with an amplitude A and a specific tone frequency $f_{tone}$, the signal $s_{bb}\left(t\right)$ can be expressed as:

(3) $$s_{bb}\left(t\right)=A\left[cos\left(2\pi f_{tone}t\right)+jsin\left(2\pi f_{tone}t\right)\right]=Ae^{j2\pi f_{tone}t}.$$

To transmit the generated signal, the instruction ‘‘BladeRF.sync_tx(buf, num_samples)’’ is employed from the Python “_bladerf” library, where “buf” represents the signal generated according to (3) and “num_samples” indicates the number of samples specified for transmission.

#### 5.4.2 Reception thread

In this thread, the instances and parameters associated with the reception are initialized. This process will operate continuously, receiving the retrieved baseband samples $s_{bb}^{\prime }\left(t\right)$ defined in (4). To facilitate the reception of the measured signal, the instruction ‘‘BladeRF.sync_rx(buf, num_samples)’’ is employed from the Python “_bladerf” library. Here “buf” denotes the size of the signal buffer $s_{bb}^{\prime }\left(t\right)$, while “num_samples” specifies the number of samples designated for reception.

#### 5.4.3 Rotation control thread

In this thread, the instances and parameters related to rotation control are initialized. This process will operate continuously, awaiting commands for calibration, clockwise movement (CW), counterclockwise movement (CCW) and movement to the −180° position. If the remainder of the division between the current angle and the angle step is zero, a trigger signal is emitted with the current angle by employing the ‘‘pyqtSignal.emit()’’ method of the “PyQt5” Python library.

#### 5.4.4 Synchronization thread

In this thread, the instances and parameters for the synchronization and signal processing are initialized. The thread will execute automatically upon pressing the “Start” button in the GUI, first sending the calibration command to the reference position, followed by the command for a full-rotation sweep. This sweep command consists of a sequence of commands for measurements ranging from −180° to 180°. Subsequently, if a trigger signal is detected at the current angle, the signal $s_{bb}^{\prime }\left(t\right)$ sampled at a sampling rate $f_{s}$ is captured and converted to power (dB) as follows

(4) $$s_{bb}^{\prime }\left(t\right)=Ae^{j2\pi f_{\text{tone}}t}+n\left(t\right)$$

where $Ae^{j2\pi f_{\text{tone}}t}$ is the signal $s_{bb}\left(t\right)$, and n(t) denotes the noise. The discrete representation of the signal $s_{bb}^{\prime }\left(t\right)$ is expressed as

(5) $$s_{bb}^{\prime }\left[n\right]=Ae^{j2\pi f_{\text{tone}}nT_{s}}+n\left[n\right]$$

with $T_{s}$ denoting the sampling period ($T_{s}=\frac{1}{f_{s}}$), and n represents the discrete-time index for each sample. The fast Fourier transform (FFT) of size M is applied to the discrete-time signal $s_{bb}^{\prime }\left[n\right]$ to convert it to the frequency domain, this is,

(6) $$S_{bb}^{\prime }\left[m\right]=\overset{M-1}{\underset{n=0}{\sum }}s_{bb}^{\prime }\left[n\right]e^{-j\frac{2\pi }{M}mn},$$

(7) $$S_{bb}^{\prime }\left[m\right]=A\overset{M-1}{\underset{n=0}{\sum }}e^{j2\pi n\left(f_{\text{tone}}T_{s}-\frac{m}{M}\right)}+\overset{M-1}{\underset{n=0}{\sum }}n\left[n\right]e^{-j\frac{2\pi }{M}mn}$$

where m represents the frequency bin. The power spectrum P[m] of the discrete signal $S_{bb}^{\prime }\left[m\right]$ is derived using

(8) $$P\left[m\right]=\frac{1}{M}{\left|S_{bb}^{\prime }\left[m\right]\right|}^{2}.$$

To mitigate the effects of noise, multiple measurements are conducted, after which the P[m] is averaged over N captures, this is,

(9) $$\bar{P}\left[m\right]=\frac{1}{N}\overset{N}{\underset{k=1}{\sum }}P_{k}^{\prime }\left[m\right]$$

Then, the bin corresponding to the strongest frequency component, $m_{\text{max}}$, is identified using

(10) $$m_{\text{max}}=\arg\max\bar{P}\left[m\right]$$

Finally, the power within that frequency range, $P_{\text{dB}}$ in (dB), is given by

(11) $$P_{\text{dB}}=10\log_{10}\left(\bar{P}\left[m_{\text{max}}\right]+ɛ\right)$$

where $ɛ=10^{-12}$ is a small constant introduced to prevent taking the logarithm of zero.

#### 5.4.5 Main thread

In this thread, the instances and parameters associated with the main program, as well as the additional process threads, are initialized. The connectivity and parameter configurations for the SDR and the stepper motor are established. Once the pair of values (θ/ϕ,dB) is obtained, it is plotted and temporarily stored in a buffer. Subsequently, the data undergoes post-processing to normalize the measurements, enhance signal quality, and calculate the critical parameters of the AUT. Normalization is conducted with respect to 0 dB. To improve the measured signal, the functions ‘‘savgol_filter’’ and ‘‘interp1d’’ from the Python “scipy” library are utilized, which serve to smooth and interpolate the data, respectively. The important parameters of the AUT are calculated using the formulas presented in Table B.1.

## 6 Operation instructions

### 6.1 Precautions for implementation

Care should be exercised when handling cables with SMA connectors, employing wide-radius bends to prevent damage. Additionally, these cables should be securely fixed in place using adhesive tape to minimize any movement that could potentially impact the measurement process. Furthermore, the measurement process should be conducted in a controlled environment, as much as possible, to mitigate any interference that may affect the accuracy of the measurements.

### 6.2 Preliminary activities

Mount and connect the reference and test antennas to the SDR using the SMA connectors, ensuring they are securely fastened to their respective support structures. Position the antennas in the appropriate orientation and polarization required for measurement. Maintain a minimum separation distance between the two antennas, determined by the frequency and length of the AUT, to facilitate measurements in the far-field region.

Power on the stepper motor controller and the Raspberry Pi. Connect the Raspberry Pi to the SDR using a USB 3.0 cable and activate the device. Launch the GUI on the Raspberry Pi by executing the main program within the Python environment, and initiate the necessary configurations.

### 6.3 Connection settings tab

**Fig. 6:**
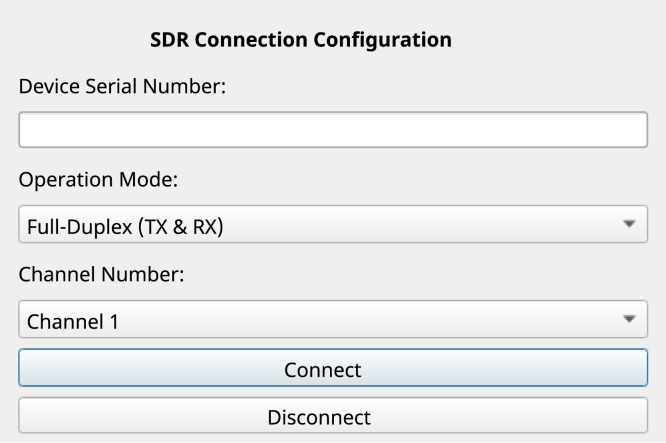
Connection settings and operating mode.

In the GUI, click the “Configuration” button to select the connection configuration options and operational mode of the bladeRF device, as illustrated in Fig. 6. For this task, enter the serial number of the bladeRF device (if available), choose the full-duplex mode of operation and select the channel for both transmission and reception (any channel may be used). Click the “Connect” button, if the connection is successful, the status will change to “Connected” and the status indicator will illuminate green. The system is now prepared for the subsequent configurations.

### 6.4 Motor control tab

**Fig. 7:**
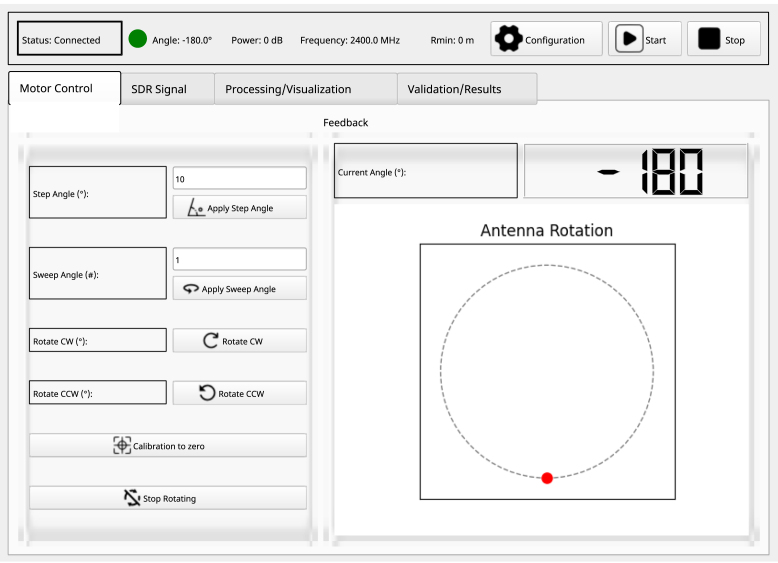
Motor control tab.

Within this tab, options for controlling and receiving feedback on the current angular position of the AUT are available, as depicted in Fig. 7. The following configurations should be considered: Enter an appropriate value based on the desired angle step accuracy. Maintain the angle sweep value at 1, unless adjustments are necessary to improve the quality of measurement; however, it is advisable to limit this to a maximum of two sweeps. If needed, utilize the “Rotate CW” and “Rotate CCW” buttons, along with the “calibration to zero” button, to manually position the antenna to the specific angle. A feedback section is provided to display the reference position of the antenna, where the red dot indicates the current angle and its orientation along the x or y axis.

### 6.5 SDR signals tab

**Fig. 8:**
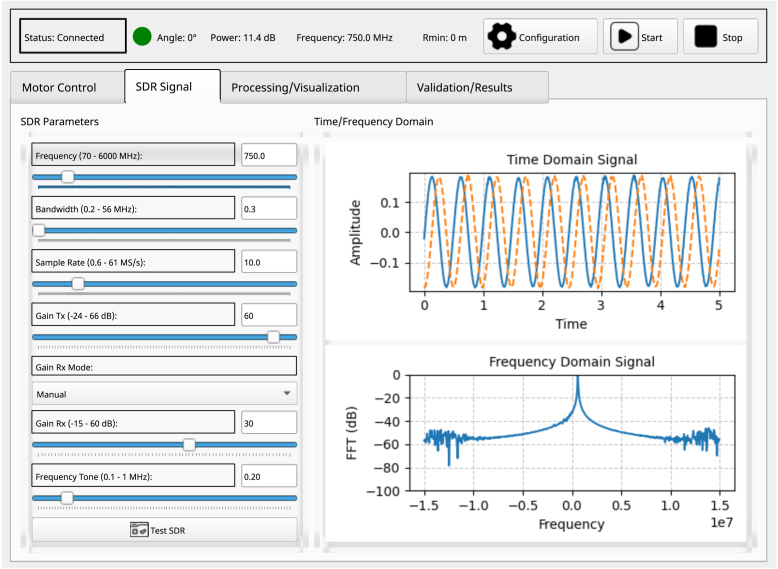
SDR signals tab in the time and frequency domain.

Within this tab, the parameters for controlling the SDR and their corresponding time and frequency domain signal graphs are presented (see Fig. 8). The following configurations should be considered: Select the desired frequency, entering a value within the range of 70 to 6000 MHz. Choose the appropriate bandwidth, or specify a narrower bandwidth to filter out interfering signals. Select the desired sampling rate; aiming for a balance between adequate resolution and manageable processing load. Determine the appropriate transmit gain or specify the maximum gain on the transmitter side to ensure proper signal transmission. For the receive gain, either select the desired value in manual mode or specify a minimum gain on the receiver side to enhance the signal-to-noise ratio (SNR).

Additionally, select the desired tone signal frequency; however, it is important to note that this selection does not impact the measurement process, and it is recommended to retain the default value. Finally, press the “SDR Test” button to activate the SDR device. A section is provided for displaying the configured signal in both the time and frequency domains, facilitating fine-tuning of the signal according to the operational frequency characteristics.

### 6.6 Processing and visualization tab

**Fig. 9:**
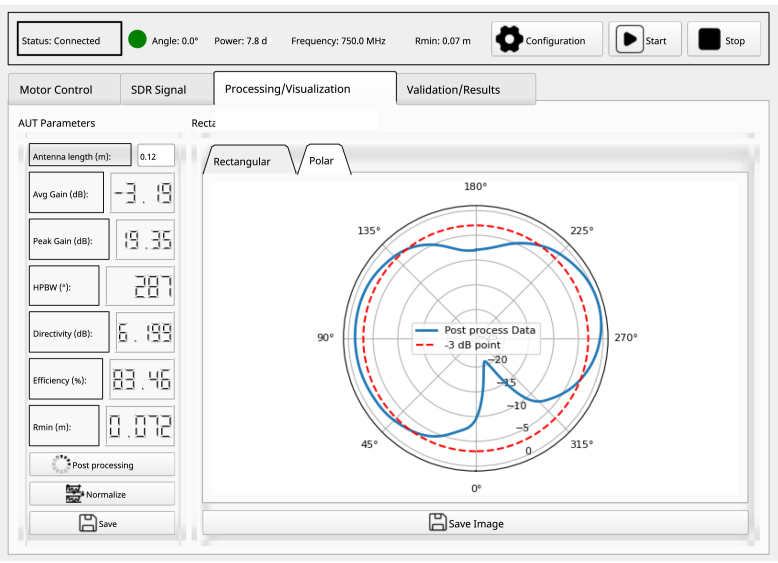
Radiation pattern processing and display tab.

Within this tab, the AUT parameters and its radiation pattern plots, presented in rectangular and polar formats, are displayed, as illustrated in Fig. 9. The following configurations should be considered: Enter the length of the AUT to establish the reference for the minimum distance between antennas (which in turn depends on the selected frequency). Click the “Start” button at the top of GUI, to initiate the measurement process in the configured plane automatically. Once the measurement is complete, press the “Normalize” and “Post-processing” buttons to enhance the quality of the measurement and to compute critical parameters such as average gain, HPBW, directivity and AUT efficiency. Finally, save the measurement data by clicking the “Save” button, ensuring that it can subsequently be compared and validated against the manufacturer’s reference data.

### 6.7 Validation and results tab

**Fig. 10:**
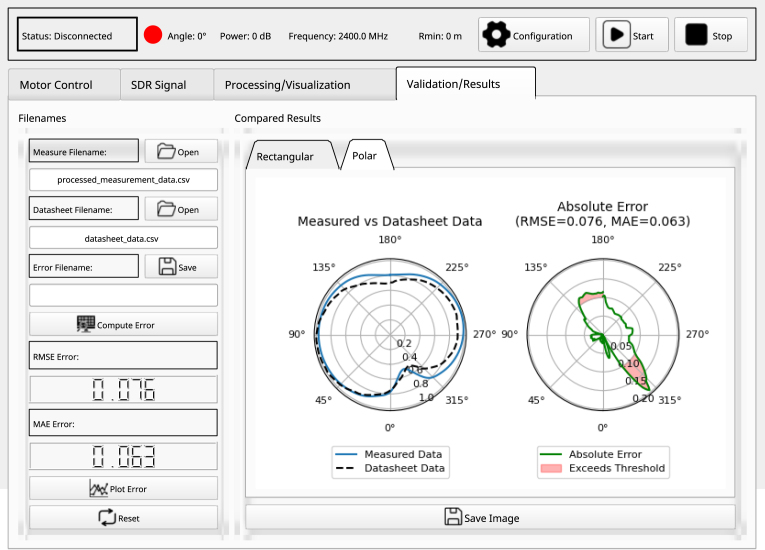
Results validation tab.

Within this tab, users can select files for validation, which include comparison and error plots of the radiation pattern presented in both rectangular and polar formats, as illustrated in Fig. 10. The following configurations should be considered: Select the files for comparison from both the enhanced measurement data and the manufacturer’s datasheet. Click the “Compute Error” button to display the absolute error in terms of mean square error and mean absolute error. Subsequently, press the “Plot Error” button to generate the comparison plot along with the absolute error in both rectangular and polar formats. Finally, users have the option to save the error data by clicking the “Save” button.

## 7 Validation and characterization

**Fig. 11:**
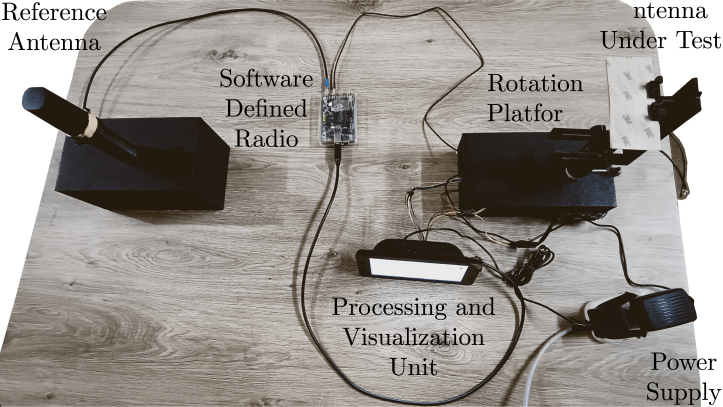
Prototype test setup.

The prototype system is designed to automatically measure the radiation pattern utilizing a SDR bladeRF for signal analysis, paired with a stepper motor-controlled rotation platform. These components are integrated into a Raspberry Pi, which serves as the processing unit. This configuration enables the accurate measurement and display of signal strength (in decibels) at various angles, based on the specified angle step, within a far-field measurement environment. The configuration of the prototype system for testing is illustrated in Fig. 11.

The components of the prototype system configuration are described as follows:

• Reference Antenna (AR): A Shockwave permanent mount external antenna operating within the frequency range of 600 to 6000 MHz.
• Antenna Under Test (AUT): A Maximus ultra-wideband flexible antenna with a frequency range of 700 to 6000 MHz.
• Rotation platform: A motor (NEMA 17) controlled by a Raspberry Pi.
• Software Defined Radio (SDR): The bladeRF 2.0 Micro xA4.
• Processing and display unit: A Raspberry Pi 4B and real-time GUI developed using the Python “PyQt5” library.
• Power supply: Provides 5 V and 12 V power for the Raspberry Pi and motor drive.

### 7.1 Performance characterization

In order to validate the prototype system, multiple signal strength measurements were conducted at each angle according to the selected angle step. These measurements were averaged and converted to decibels. To obtain the data required for plotting the radiation pattern, it was necessary to perform a complete sweep encompassing one revolution (360°).

#### 7.1.1 Measurements

**Fig. 12:**
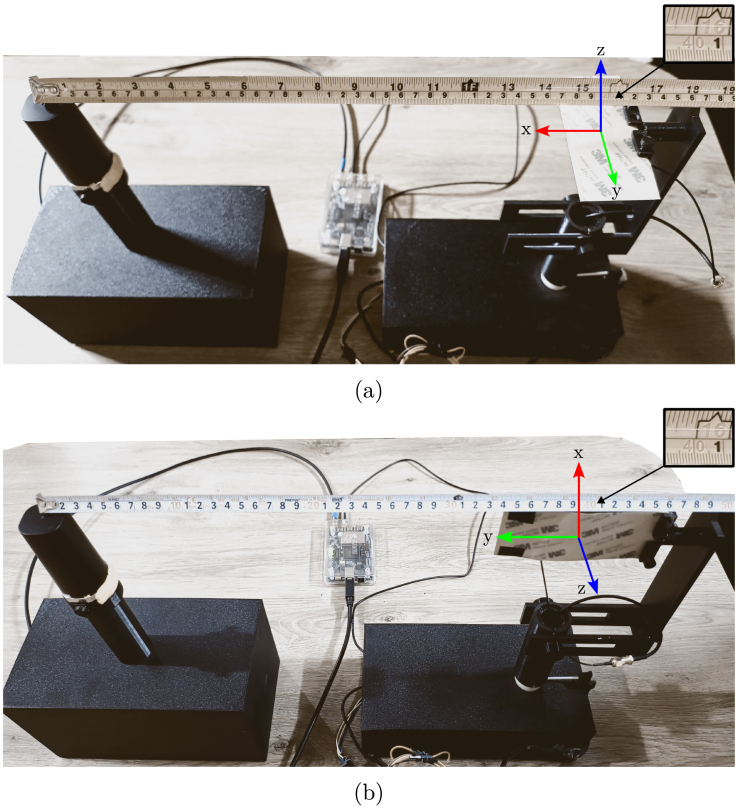
Setup for far-field measurements: (a) horizontal and (b) vertical.

Considerations for measuring the radiation pattern of the AUT are outlined as follows:

• Measurements were conducted at frequencies of 750 MHz and 2600 MHz.
• Data acquisition occurred in both the horizontal and vertical planes, utilizing an angle step of 10°.
• The AUT measurements satisfied the far-field conditions within a non-anechoic environment and were recorded for both horizontal and vertical polarization, as depicted in Fig. 12.
• This far-field condition is setup with the follow geometry: L=0.12m, λ=0.400m,0.115m (for each operational frequency), giving a $R_{min}=0.07\;m$ for 750 MHz and $R_{min}=0.25\;m$ for 2600 MHz. Therefore, the setup show in Fig. 12, with R=0.40m is inside of far-field region.

#### 7.1.2 Results

**Fig. 13:**
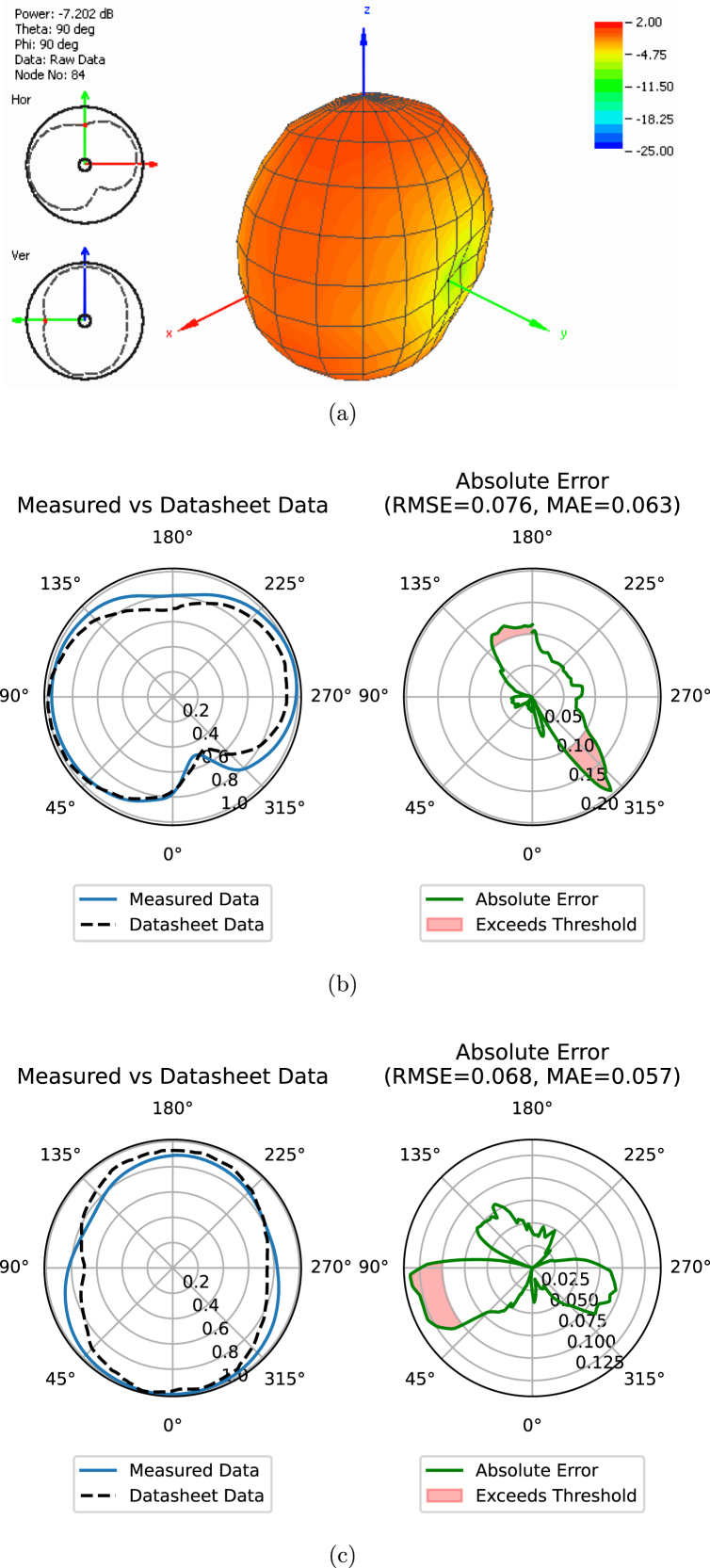
(a) Radiation patterns of the manufacturer’s specifications and the prototype system in the (b) horizontal and (c) vertical planes at 750 MHz, presented in polar form.

**Fig. 14:**
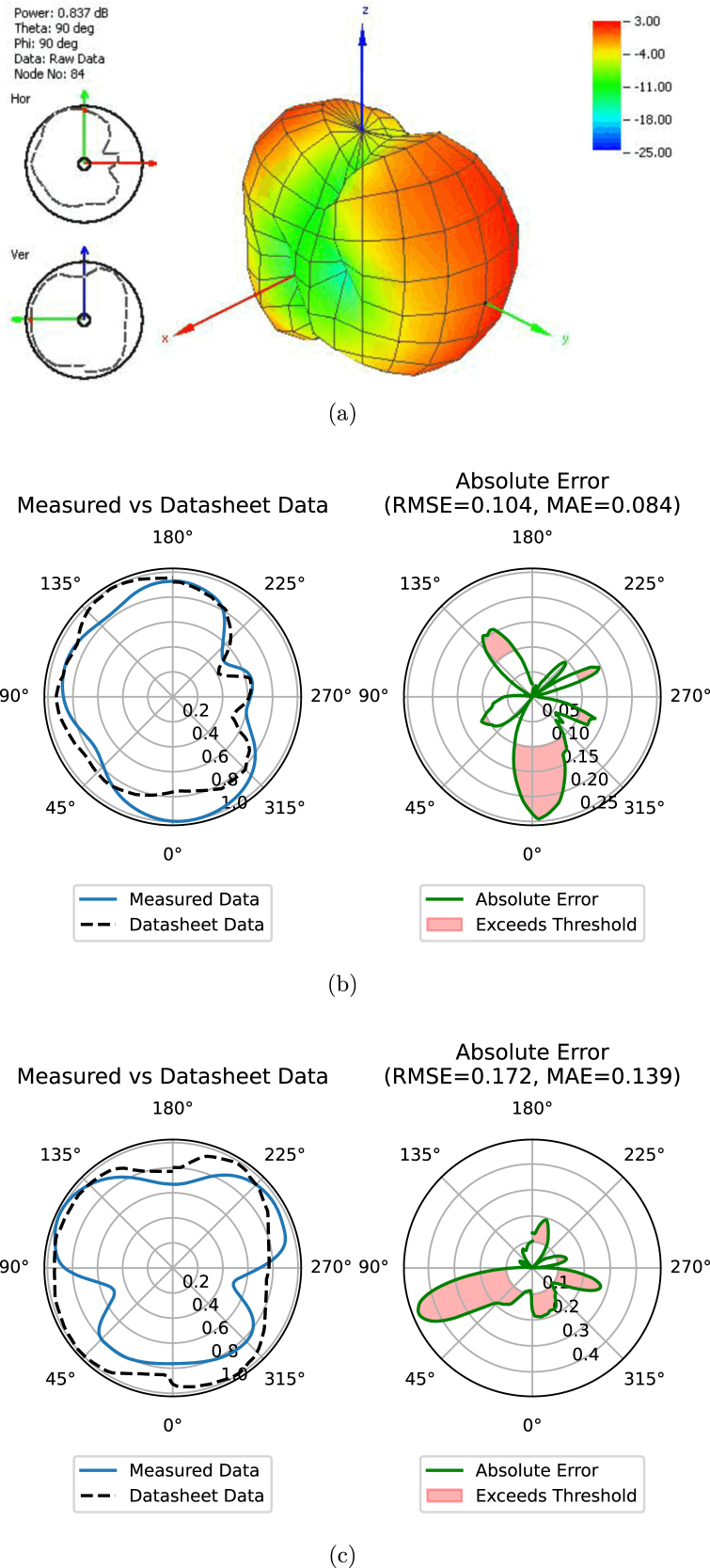
(a) Radiation patterns of the manufacturer’s specifications and the prototype system in the (b) horizontal and (c) vertical planes at 2600 MHz, presented in polar form.

The radiation pattern measurements obtained from the prototype system were compared with the manufacturer’s datasheet, found in [17]. This comparison was conducted for the frequency of 750 MHz, illustrated in Fig. 13, and for 2600 MHz, as shown in Fig. 14. The results of this comparison are presented in Table 4, demonstrating the performance of the prototype system. Notably, the radiation pattern measurements obtained with the prototype closely resemble those provided in the manufacturer’s datasheet.

To quantify the accuracy of the measurements, the RMSE and MAE errors were computed using the formulas outlined in Table C.1. For the signal frequency of 750 MHz, the absolute errors were as follows: an RMSE of 0.076 (1.462 dB) and an MAE of 0.063 (1.226) in the horizontal measurement, and an RMSE of 0.068 (1.410 dB) and an MAE of 0.058 (1.200 dB) in the vertical measurement. For the signal frequency of 2600 MHz, the absolute errors were: an RMSE of 0.104 (1.165 dB) and an MAE of 0.084 (0.937 dB) in the horizontal measurement, and an RMSE of 0.172 (3.260 dB) and an MAE of 0.139 (2.625 dB) in the vertical measurement.

These error values indicate that the prototype system provides a close approximation to the expected antenna performance, aligning with commercial accuracy standards of ±1dB, such as those of the MegiQ RMS-0460 system, particularly considering that the tests were conducted outside of an anechoic chamber.

**Table 4:** Result of the absolute error in the measurements.

Measurement plane	Frequency (MHz)	RMSE (dB)	MAE (dB)
Horizontal	750	0.076 (1.462)	0.063 (1.226)
(Azimuth)	2600	0.068 (1.410)	0.057 (1.200)
Vertical	750	0.104 (1.165)	0.084 (0.937)
(Elevation)	2600	0.172 (3.260)	0.139 (2.625)

Although repeatability was not explicitly quantified, consistent results were demonstrated through antenna characterization performed over multiple full-rotation sweeps, indicating good repeatability performance.

### 7.2 Capabilities and limitations

#### 7.2.1 Capabilities

The prototype system possesses the following capabilities:

• Automatic measurement of a complete 360° sweep with adjustable angle step resolution.
• Real-time visualization of measured data in both rectangular and polar formats.
• Capability to measure radiation patterns in both horizontal and vertical orientations.
• Measurement frequency range spanning from 70 MHz to 5.9 GHz.

#### 7.2.2 Limitations

The prototype system exhibits the following limitations:

• As illustrated in Fig. 13, Fig. 14, measurement errors were observed due to the differing conditions under which the measurements were compared to those specified by the manufacturer. Consequently, the performance of the measurements obtained with the prototype system is influenced by environmental electromagnetic interference.
• Manual adjustments are required for measurements conducted in both the horizontal and vertical planes, as the prototype system is equipped with only a single stepper motor.
• Manual adjustment of the SDR signal parameters is necessary to align with the designated frequency and calculated minimum distance.

Furthermore, SDR devices have inherent limitations when measuring radiation patterns compared to VNAs. Firstly, the noise floor is higher, which means that weak signals in the radiation pattern may be masked by noise, especially a problem when measuring null depths or low sidelobes. Secondly, the dynamic range is lower, which affect the accuracy of radiation pattern measurements, particularly when the antenna exhibits a high dynamic range, as strong signals may saturate the receiver and weak signals may fall below the noise floor. Lastly, although it may not be critical for radiation pattern measurement, the phase accuracy is also lower.

### 7.3 Application

The prototype system developed in this work has numerous applications in the research, design, and evaluation of antennas operating at sub-6 GHz frequencies. It is well-suited for academic environments, facilitating practical studies of electromagnetic radiation. Additionally, the system can be utilized within the telecommunications industry for antenna design, validation, and various applications. Its capability to function effectively in open environments, without the necessity of anechoic chambers, enhances its practicality in real-world testing scenarios, particularly when compared to high-end testing equipment such as VNAs.

### 7.4 Future developments

To improve usability compared with commercial systems and address current limitations, the following future works are planned:

• Test condition: The prototype will be used to evaluate a custom-designed antenna and validate its simulated radiation pattern. This enables direct, consistent measurements within the same system, minimizing errors caused by differing conditions in commercial or manufacturer test environments.
• Scanning the horizontal and vertical planes: In the prototype system, a second stepper motor will be integrated to scan the vertical plane. This will be in addition to the stepper motor that already permits scanning of the horizontal plane. This involves redesigning the adjustment mechanism to enable automatic 3D radiation patterns.
• Signal parameter automation: In the prototype system, an algorithm will be used to automatically configure SDR signal parameters (mainly Rx and Tx gain) based on the operational frequency, the measured or estimated distance between the antennas, and the real-time received signal strength.
• Accommodating custom antenna designs: In the prototype system, a modular adjustment mechanism will be integrated to support a range of custom or experimental antennas, both for the AR and AUT. This will allow the system to adapt to antennas with diverse physical dimensions, mounting requirements, and radiation characteristics, such as polarization, beamwidth, and frequency range.
• Compatibility with additional SDRs: The prototype system can be upgraded with an additional SDR to enable more advanced configurations, such as multiple input-multiple output (MIMO) antenna setups. This expansion increases the system’s usefulness for more complex antenna characterization tasks, including beamforming experiments, channel sounding and spatial diversity analysis.

### 7.5 Conclusion

The developed prototype system demonstrates the feasibility of a low-cost, automated solution for antenna characterization. By integrating an SDR (bladeRF micro xA4), a rotation platform driven by a stepper motor and controlled by a Raspberry Pi 4B, and a Python-based graphical user interface (GUI), the system successfully executed 360° sweeps of the radiation patterns enabling real-time visualization and data recording.

The results indicate an acceptable margin of error relative to the manufacturer’s reference data, with RMSE values of less than 0.172 (3.260 dB) and MAE values of less than 0.139 (2.625 dB). These findings validate the system’s accuracy, achieved without the use of an anechoic chamber.

The prototype serves as a robust foundation for future enhancements, such as automating horizontal and vertical plane scanning without manual adjustments, accommodating custom antenna designs, and ensuring compatibility with additional SDRs. Its modular and open-source architecture allows for modification and adaptation, making it suitable for a variety of practical research applications within the telecommunications field.

## CRediT authorship contribution statement

**Alex Kana-Chuctaya:** Writing – original draft, Visualization, Validation, Software, Methodology, Formal analysis. **Alexander Hilario-Tacuri:** Writing – review & editing, Supervision, Resources, Project administration, Investigation, Conceptualization.

## Declaration of competing interest

The authors declare that they have no known competing financial interests or personal relationships that could have appeared to influence the work reported in this paper.

## Figures and Tables

**Table A.1:** Specifications of SDRs for selection purposes.

Specs	Lime SDR Mini	bladeRF 2.0 Micro	Ettus USRP B210	PlutoSDR
Frequency range	10–3500 MHz	47 MHz–6 GHz	70 MHz–6 GHz	325–3800 MHz
Sampling rate	30.72 MSPS	61.44 MSPS	61.44 MSPS	7.5–12 MSPS
RF bandwidth	30.72 MHz	200 kHz–56 MHz	200 kHz–56 MHz	20 MHz
Tx/Rx channels	1/1	2/2	2/2	1/1
ADC/DAC resolution	12	12	12	12
Transmission power	–	8 dBm (CW)	10 dBm	–
Temperature range	–	0–70 °C	25 °C	10–40 °C
Size	9.5 cm × 3.6 cm × 1.3 cm	11,1 cm × 7,3 cm × 2,4 cm	15.5 cm × 9.7 cm × 1.5 cm	–
Weight	42 g	125 g	350 g	127 g
Approximate price	299 USD	480 USD (xA4) 720 USD (xA9)	1200 €	140 €

**Table B.1:** Definition of key parameters for antenna characterization.

Parameter	Formula	Description
Average gain	$G_{\text{avg}}=10\log_{10}\left(\frac{1}{2\pi }\int_{0}^{2\pi }P\left(\theta \right)\;d\theta \right)$	The mean gain in (dB) over a 360° azimuth, is given by normalized power pattern P(θ).
Peak gain	$G_{\text{peak}}=\max_{\theta }P_{\text{dB}}\left(\theta \right)$	This shows the maximum gain of the AUT in (dB) over all measured angles.
Half-Power beamwidth		
(HPBW)	$\text{HPBW}=\theta_{2}-\theta_{1}$	The angular width (°) is the distance between the points where the gain drops 3 dB below $G_{\text{peak}}$.
Directivity	$D=\frac{4\pi \cdot G_{\text{peak,linear}}}{P_{\text{rad}}}$ $D_{\text{dB}}=10\log_{10}\left(D\right)$	Directivity in (dB) is based on peak gain and total radiated power $P_{\text{rad}}$.
Efficiency	$\eta =\frac{G_{\text{avg}}}{G_{\text{peak}}}\cdot 100\text{\%}$	The ratio between the average and peak gains, expressed in (%).

**Table C.1:** Definition of error parameters: MAE and RMSE.

Parameter	Formula	Description
MAE (Mean Absolute Error)	$\text{MAE}=\frac{1}{n}\sum_{i=1}^{n}\left|y_{i}-{\overset{ˆ}{y}}_{i}\right|$	Average of the absolute differences between the measured and expected values.
RMSE (Root Mean Square Error)	$\text{RMSE}=\sqrt{\frac{1}{n}\sum_{i=1}^{n}{\left(y_{i}-{\overset{ˆ}{y}}_{i}\right)}^{2}}$	Square root of the average of the square of the differences between the measured and expected values.

## Appendix A Hardware selection

This appendix presents a comparative analysis of the specifications of several SDRs as detailed in Table A.1.

## Appendix B Antenna parameters

This appendix presents the formulas for calculating the key parameters of the AUT antenna, as summarized in Table B.1. The radiation power is computed using $P_{\text{rad}}\approx \sum_{i=0}^{N}P\left(\theta_{i}\right)\cdot \Delta \theta$, where N represents the number of measured angles and Δθ is the angular step in radians [2], [16], [18].

## Appendix C Absolute error calculation

This appendix presents Table C.1, which demonstrates the application of error parameters for validating the prototype system. In Table C.1, $y_{i}$ denotes the reference value, ${\overset{ˆ}{y}}_{i}$ represents the measured value and n indicates the total number of samples [19].
